# Mechanics of small intestine motility for oral macromolecular delivery: modelling segmentation versus peristalsis

**DOI:** 10.1080/10717544.2025.2607779

**Published:** 2025-12-24

**Authors:** Benyamin Naranjani, Shakhawath Hossain, Marco Tjakra, Pardis Azhand, Christel Bergström, Patrick Sinko, Per Larsson

**Affiliations:** a Department of Pharmacy, Uppsala Biomedical Center, Uppsala University, Uppsala, Sweden; b Swedish Drug Delivery Center (SweDeliver), Uppsala University, Uppsala, Sweden

**Keywords:** Biologics, oral drug delivery, oral insulin, intestinal motility, peristalsis, segmentation, permeation enhancer, computational fluid dynamics, macromolecular transport, machine learning

## Abstract

Intestinal motility, including peristalsis and segmentation, drives complex fluid movements critical for the oral delivery of biologics and other macromolecules. Despite advances, oral delivery remains commercially limited by low bioavailability, often attributed to poor epithelial permeability. However, variability in motility patterns may also play a critical role, influencing intraluminal distribution and thus absorption, yet this aspect remains underexplored. Here, we combine computational fluid dynamics and machine learning to evaluate how motility type, intensity, pocket size, contractility, and fluid composition affect the delivery of a model macromolecule (insulin) and a permeation enhancer (sodium caprate, C10). We find that segmentation, especially at light intensity, consistently enhances epithelial colocalisation over peristalsis. Under segmentation, smaller pocket sizes (2 mL versus 10 mL) and stronger contractility (occlusion ratio 0.3) yielded optimal performance. Our extreme gradient boosting regression model identified pocket volume, contractility, and motility type as dominant predictors of colocalisation. In a comparative analysis, segmentation led to 128% and 137% higher maximum normalised concentrations of insulin and C10, respectively, than moderate peristalsis with a nutritional drink. Overall, segmentation achieved 6.7-fold and 8.0-fold higher average maximum normalised concentrations for insulin and C10, respectively. These results emphasise segmentation, characteristic of the fed state, as a superior motility pattern for macromolecular absorption compared to peristalsis during the migrating motor complex (MMC). By elucidating the interplay between motility and transport, our findings may guide the design of more effective oral formulations and support personalised strategies for drug delivery based on individual motility profiles.

## Graphical abstract

**Figure:**
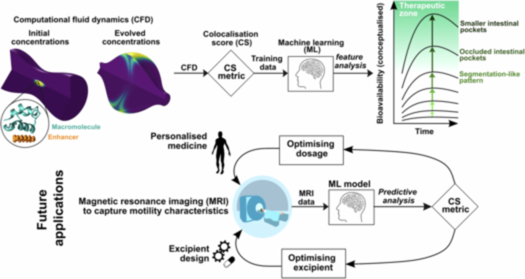

## 1. Introduction

Oral delivery of biologics presents a promising avenue for therapeutic administration (Lau and Dunn 2018; Wang et al. 2022), but is associated with challenges, due to the complexities of the gastrointestinal tract. These challenges include susceptibility to inactivation of the active substance due to extreme pH values (Hersey and Sachs 1995), enzymatic degradation, and poor permeability of the intestinal epithelium. As a result, the oral bioavailability of therapeutic macromolecules (MMs) is typically <1% (Carino et al. 2000; Fonte et al. 2013; Pridgen et al. 2014), severely limiting their therapeutic efficacy when administered orally. One approach to increase the oral bioavailability of MMs involves the use of permeation enhancers (PEs), which transiently increase the permeability of the intestinal epithelium by altering tight junction integrity or membrane fluidity, thereby facilitating the absorption of MMs. Examples include sodium caprate (C10) (Lo and Huang 2000; Shanmugam et al. 2013; Ates et al. 2016; Maher et al. 2021), sodium salcaprozate (SNAC, used in the development of oral semaglutide) (Buckley et al. 2018; Lewis et al. 2022), and sodium caprylate (C8, used in oral octreotide) (Biermasz 2017; Brayden and Maher 2021).

Motility is intrinsic to the digestive and absorptive mechanisms of the GI tract, and disruptions can lead to clinical complications such as impaired feeding tolerance, malnutrition, gastroesophageal reflux, dyspeptic symptoms, irritable bowel syndrome (Khan and Chang 2010), small intestinal bacterial overgrowth, and weight loss (Ladopoulos et al. 2018; Pimentel et al. 2020; Nightingale et al. 2020). Motor dysfunction has also been observed in chronic intestinal pseudo-obstruction (De Giorgio et al. 2011). Alterations in the contractile behaviour of the circular and longitudinal smooth muscles coupled with variations in fluid rheology significantly influence flow patterns, luminal pressure, peak velocity, and the extent of shearing and mixing (Yamaguchi et al. 2018; Avvari 2019). Understanding these influences is critical for the optimal design of pharmaceutical dosage forms for oral administration, as the mechanical and hydrodynamic environment within the GI tract affects drug dissolution, transport, and absorption.

The motility patterns in the small intestine originate from activation of myogenic and neural regulatory mechanisms (Wingate 1983; Huizinga et al. 1998), resulting in complex contractions that impact drug delivery. In the fed state, non-propagating circumferential or segmental contractions rhythmically squeeze the ingesta, causing it to slosh back and forth locally, which enhances mixing with digestive enzymes and facilitates nutrient absorption (Melville et al. 1975). In the fasted state, propagating circumferential and longitudinal contractions, known as peristaltic contractions, propel intraluminal content along the length of the intestine (Hennig et al. 1999; Whitehead 2001; Bratten and Jones 2007; Cheng et al. 2010; Khurana et al. 2019).

Several studies have previously explored segmental activity in the small intestine through various approaches. Experimental studies have shown that elevated osmolarity of saline and increased energetic load of nutrients and decanoic acid (1 mM) can elicit a transition from propulsive to segmenting motility patterns (Schemann and Ehrlein 1986; Schmid and Ehrlein 1993; Huizinga et al. 2014). *In vitro* experiments by Tharakan et al. replicated segmentation by using inflatable cuffs to compress or decompress the inner intestinal tube (Tharakan et al. 2010). Computational simulations by de Loubens et al. using a lattice-Boltzmann numerical method found that pendular activity enhances *in situ* mixing of viscous fluids and accelerates diffusive mass transfer, with segmentation playing a crucial role in mixing particulate suspensions with high solid volume ratios (de Loubens et al. 2013). Chambers et al. developed a computational model to investigate the mean neuron firing rate, simulating the feedforward ascending and descending reflex pathways in the guinea pig small intestine (Chambers et al. 2008).

However, while these studies have advanced our understanding of segmentation, they did not address how varying physiological conditions affect the mechanisms of segmentation and their impact on luminal transport processes. Moreover, there remains a need to understand how segmentation influences drug transport under different physiological conditions to inform the design of effective oral drug delivery strategies. Importantly, although fasted-state peristalsis, referred to as the migrating motor complex (MMC), is the predominant focus in oral peptide development, this does not necessarily reflect the conditions under which optimal absorption occurs. Biologics such as oral semaglutide, co-formulated with SNAC, are absorbed predominantly in the stomach rather than the small intestine, and therefore rely on strict fasted-state dosing instructions to stabilise a narrow gastric absorption window rather than to facilitate intestinal delivery.

This creates an apparent discrepancy with clinical observations. While fasted-state dosing is required for drugs that target gastric uptake, many macromolecular formulations under development aim for absorption in the small intestine, where fed-state segmentation governs intraluminal mixing and can enhance local drug-epithelium interactions. Yet the influence of fed-state motility patterns, particularly segmentation, on the transport of MMs and PEs remains insufficiently characterised. Addressing this gap is essential for advancing formulations intended for small-intestinal absorption and for understanding inter-individual variability in patients with altered motility patterns, such as those with IBS or diabetes, where segmentation dynamics may differ from healthy controls.

In a previous study, we used computational fluid dynamics (CFD) simulations to model peristaltic motility within a segment of the small intestine, revealing that a reduction in peristaltic wave speed and an increase in contractility of intestinal water pockets were associated with higher concentrations of MM and PE at the epithelial surface. This demonstrated how CFD can be implemented to predict the impact of specific motility parameters on drug transport, thereby informing the design of dosage forms that are optimised for certain physiological conditions.

Building on this approach, in the current study, we focus on a model that replicates a segment of the small intestine that is undergoing intestinal segmentation. Our model is based on observed human parameters and includes intestinal pocket volumes of 2 mL and 10 mL with wavelengths of 2.4 cm and 4.1 cm, and occlusion ratios (OR) from 0.3 to 0.7, reflecting statistically significant variations found in humans (Lindahl et al. 1997; Clarysse et al. 2009; Mudie et al. 2014; Huizinga et al. 2015). We developed a mathematical model for segmentation flow based on videofluoroscopy of the small intestine to approximate segmentation motility in our simulations (Ehrlein et al. 1987). To the best of our knowledge, this is the first model that specifically simulates intestinal segmentation with a focus on its impact on the transport of MMs and PEs.

We used CFD to investigate the influence of recursive segmental contractions on the generated intestinal fluid structures, shear stress levels, and luminal velocity magnitude. To attain an overall assessment of the intraluminal transport capabilities of segmental motility, we performed a series of investigations on how variability in motility intensity, contractility levels, pocket volume, and fluid viscosity impacts intestinal transport. In addition, we compared segmentation motility to peristaltic motility to assess the efficacy of drug absorption between the two modes of motility. We then investigated the intraluminal transport of insulin and C10, the model MM and PE respectively, to determine what factors yield the optimum motor activity for the transport of molecules released from a dosage form to the epithelial surface. We propose a colocalisation scoring metric that considers spatiotemporal factors for the optimal coadministration of MMs and PEs. To analyse the impact of these variability factors on colocalisation, we employed an extreme gradient boosting (XGBoost) regression model, which allowed us to identify the most influential factors affecting colocalisation. Our goal was to uncover and quantify the characteristics of optimal intestinal motor activity for effective delivery of drug molecules to the epithelium. Our findings have implications for excipient design in drug development, as understanding the factors influencing drug transport can inform the creation of formulations that enhance delivery outcomes. Additionally, this work lays the groundwork for personalised medicine applications, where individual variations in intestinal physiology and motility patterns could be considered to optimise oral drug delivery strategies tailored to each patient.

## 2. Methods

### 2.1. Problem description and assumptions

The intestinal segment model comprised three pockets for segmentation, each exhibiting segmental motility, and the peristalsis model comprised 30 pockets in length to ensure the drug molecules remained within the computational domain throughout the simulation. For both motility types, simulations were conducted over 30 periods.

To model the recursive contractions and expansions of the epithelium during segmentation, we prescribed the wall velocity as:

(1) $$U_{r}\left(Z,t\right)=2\pi \frac{\delta }{t_{p}}\;\cos\left(2\pi \frac{Z}{\lambda }\right)\cos\left(2\pi \frac{t}{t_{p}}\right)+f\left(t\right)$$

where f(t) is a correction term introduced to ensure strict conservation of luminal volume and to eliminate non-physical suction or ejection of intestinal fluid during wall motion.

To determine this correction term, we imposed a mathematical constraint requiring zero net volumetric change during each segmentation cycle:

(2) $$\mathrm{dv}=\int_{0}^{\lambda }2\pi G\left(Z,t\right)U_{r}\left(Z,t\right)\mathrm{dtdz}=0$$

where G(Z,t) denotes the instantaneous radial position of the epithelial boundary. Substituting equation (1) into equation (2), expressing accordingly, and simplifying yields an ordinary differential equation whose solution provides the analytical form of the correction term:

(3) $$f\left(t\right)=\frac{-\frac{\pi \delta^{2}}{t_{p}}\sin\left(4\pi \frac{t}{t_{p}}\right)}{2\sqrt{r_{0}^{2}+\frac{\delta^{2}}{4}\left(\cos\left(4\pi \frac{t}{t_{p}}\right)-1\right)}}$$

This ensures that wall deformation remains periodic while maintaining exact volume conservation throughout the cycle. Full mathematical derivation is provided in Appendix A.

We examined drug release at two positions: the most and least occluded positions. For both segmentation and peristalsis simulations, we used initial concentrations of 2 mM for insulin and 100 mM for C10. These simulations allowed for a direct comparison between peristaltic and segmental motility in terms of their efficacy in transporting molecules to the epithelial surface.

Both Newtonian and non-Newtonian fluid behaviours were considered by inputting the rheological properties of water and nutritional fluid to represent intestinal fluids in the fasted and fed states (Appendix D). The intestinal epithelium was modelled as an impermeable wall boundary with a no-slip condition.

### 2.2. Computational fluid dynamics (CFD)

We performed transient 2D-axisymmetric simulations using COMSOL Multiphysics 6.1, a finite-element solver to simulate the conjugated fluid flow and mass transfer in an intestinal model. We implemented a laminar flow solver to solve the Navier-Stokes equations for an incompressible fluid in the absence of body forces. The Navier-Stokes solver was integrated with a mass transfer module using the velocity components as inputs to derive the time-dependent concentration of molecules.

Intestinal motility was represented by a series of waves. For the flow solver, we applied periodic boundary conditions at the inlet and outlet to replicate this cyclic behaviour, while open boundary conditions were implemented for the mass transfer module to facilitate mass escape. A moving mesh interface was incorporated to accommodate changes in the mesh configuration, aligning it with the deformations in the intestinal wall boundary.

To ensure the accuracy of the generated mesh, we carried out a grid sensitivity analysis, comparing velocity components in sensitive regions and the average insulin concentration at the epithelial surface for the fluid flow and mass transfer solvers (Appendix E). The mesh was refined near the epithelial surface and in regions with high velocity gradients to capture the detailed flow and mass transfer phenomena.

To model peristalsis, we performed simulations in the moving frame of reference, thereby eliminating the need for the implementation of a moving mesh. The velocity field was then recovered in the actual fixed frame for the streamline figures by subtracting the wave propagation speed from the fluid velocity.

### 2.3. Rheometer measurements

An ARES-G2 from TA Instruments was used to measure the shear dependence on the dynamic viscosity of the nutritional drink (Nestle Resource MiniMax, Figure S4). The sample volume was 10 mL to ensure optimal coverage of the geometry. Before each replicate measurement, the torque and zero positions of geometries were tared. Frequency sweep mode measurements, ranging from 0.01 s^−1^ to 300 s^−1^ using a double gap concentric cylinder geometry were performed at 37 °C. A 60 s soak time was used to equilibrate the temperature across the samples and geometries. Five sampling points per logarithmic decade were captured during sweeps. All data were collected and plotted as inputs for the simulation.

### 2.4. Density measurements

The fluid density was measured using two orthogonal methods: a pycnometer and a tensiometer (Supplementary Figure S5). For the pycnometer (BRAND BLAUBRAND) measurements, the container and sample mass were weighed on a Sartorius ENTRIS 224i-1s electronic balance. Density was calculated based on the volume of the calibrated vessel (5.316 cm3) at 20 °C. For the tensiometer (Attension Sigma 703D) measurement, a density probe (a smooth glass sphere) was immersed in a small container with the fluid of interest. The sample was preheated to 38 °C and quickly transferred to the sample stage of the tensiometer for measurement. The probe was then inserted slowly and centred in the sample container away from the container edges and the liquid surface. The density was automatically calculated using a tensiometer based on buoyancy principles.

### 2.5. Statistical analysis

To determine the appropriate statistical tests for evaluating the data, we first assessed the normality of the data distribution. Normality was evaluated using Q-Q (Quantile-Quantile) plots, which visually compare the quantiles of the sample data against those of a normal distribution. The Q-Q plots for each comparison group are provided in the supplementary materials (Appendix F). Given that the data deviated from normality in many cases, we opted to use non-parametric tests. Specifically, we employed Dunn's multiple comparisons test, the Mann-Whitney U test, or the Kruskal-Wallis test, as appropriate, to determine statistical significance. Significance levels are indicated by asterisks, where *, **, ***, and **** denote *p*-values of < 0.05, < 0.01, < 0.001, and < 0.0001, respectively.

### 2.6. Machine learning analysis

To further analyse the impact of variability factors on the colocalisation score (CS), we employed an XGBoost regression model. The dataset consisted of all simulated cases within Cluster 3 identified by K-means clustering. The target variable was the CS, which is analytically bounded between 0 and 1. Prior to model training, all descriptor values were scaled using Min–Max scaling to the [0, 1] range. Min–Max scaling was chosen to place all inputs on a consistent numerical domain, support stable optimisation during gradient-boosting, and facilitate SHAP value interpretation on a comparable scale. Categorical variables were encoded using one-hot encoding to convert them into a suitable numerical format without introducing unintended ordinal relationships.

We split the dataset into training and testing sets using an 80/20 partition. Hyperparameter tuning was performed using GridSearchCV with 3-fold cross-validation, exploring a focused yet sufficiently expressive grid of hyperparameters to optimise the model. A 3-fold scheme was selected to balance variance and bias while maintaining adequate sample size in each fold, which is essential given the modest dataset generated from the CFD simulations. The search included ranges for the number of estimators (50–700), learning rate (0.005–0.2), maximum depth (3–9), subsample ratios (0.6–1.0), and regularisation parameters.

To interpret the model and identify the most impactful features, we utilised SHAP, which quantifies the contribution of each feature to individual model predictions. SHAP values were computed for all features, and the mean absolute SHAP values were used to rank their relative importance.

## 3. Results

### 3.1. Fluid dynamics and shear stress

#### 3.1.1. Motility waveform factors that influence the evolution of concentration transport to the epithelium

**Figure 1.**
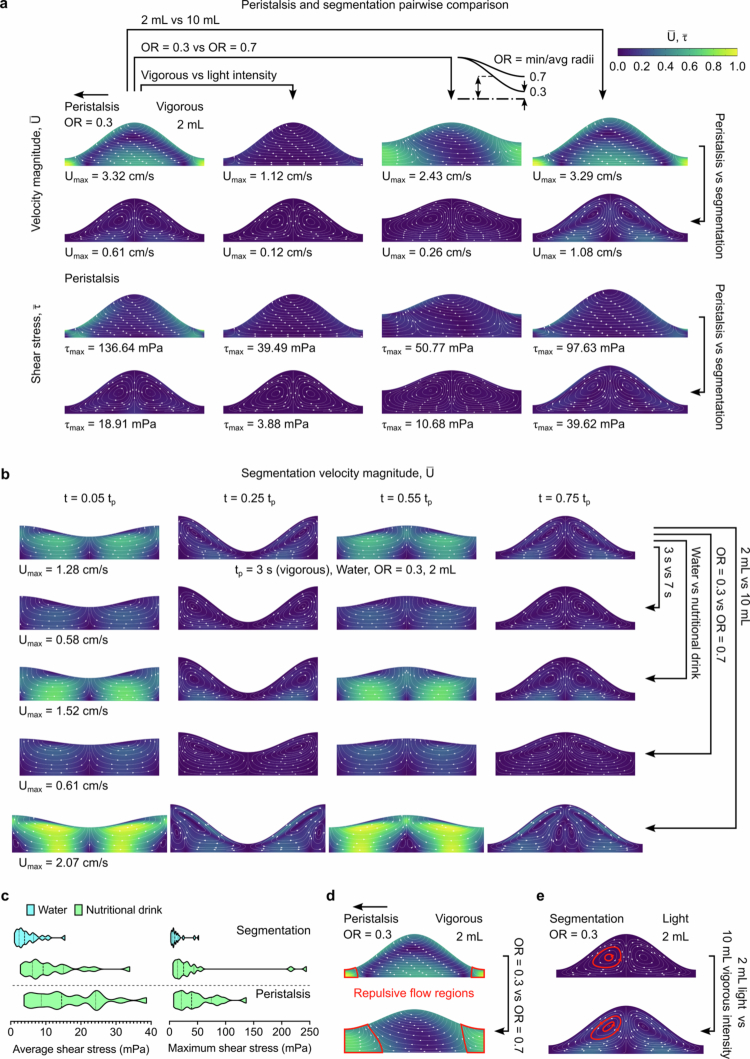
CFD simulations showing intraluminal fluid structures and flow dynamics under different motility conditions. **a** Comparative representation of the streamlined velocity magnitude and shear stress contours for intestinal pockets undergoing peristalsis and segmentation. **b** Evolution of fluid flow patterns during segmentation, illustrating differences in axial transport and circulation zones within the intestinal lumen. **c** Multifactor analysis of average and maximum shear stress along the epithelial surface, showing distributions of the mechanical forces at the epithelial surface for segmentation and peristalsis with water (blue) or nutritional drink (green) as the intestinal fluid. **d** Comparison of the repulsive flow regions for peristalsis with OR = 0.3 versus 0.7. **e** Collective impact of pocket size and motility intensity on the topology of the vortices and distribution of shear stress. The motility parameters used in these simulations, including wave speed, OR, segmentation wavelength and pocket volume, fall within the ranges reported for human small-intestinal motility in videofluoroscopy and manometry studies (Ehrlein et al. 1987; Lindahl et al. 1997; Clarysse et al. 2009; Mudie et al. 2014; Huizinga et al. 2015).

We studied the intraluminal transport of dissolved molecules, using CFD to investigate the impact of motility type (segmentation and peristalsis) and its intensity on drug delivery to the epithelium (see Appendix A for detailed segmentation modelling). To this end, we modelled three wave speed rates which approximate different motility intensity levels: light (7 s and 0.5 cm/s), moderate (5 s and 1 cm/s), and vigorous (3 s and 1.5 cm/s). We hypothesised that these patterns are periodic; however, it is important to acknowledge that perfect patterns may not occur *in vivo*. As segmentation primarily occurs in the fed state in adults, we examined two types of intestinal fluid composition, water and a nutritional drink supplement. To account for the variability that is observed in blood plasma concentration profiles we explored variations in contractility (OR = 0.3, 0.5, and 0.7, i.e. from most to least occluded), and intestinal pocket volume (2 mL and 10 mL) on the generation and evolution of intraluminal fluid structures and shear stress levels in the epithelium (Figure 1a–d). Finally, we compared peristalsis and segmentation waveforms which are observed in different phases of the MMC and during the fed state.

Peristalsis generates fluid structures that predominantly facilitate axial transport in the direction of the propagating wave (Shapiro et al. 1969; Pozrikidis 1987; Naranjani et al. 2023). This phenomenon was accompanied by the emergence of repulsive flow regions, where the epithelial surface exhibited increased occlusion, as shown in our simulations (Figure 1a,d). These regions displayed higher velocity magnitudes, with their sizes expanding in areas with diminished contractility (OR = 0.7). Conversely, segmental motility induced a different flow pattern, marked by the formation of two localised counterrotating circulation zones within each water pocket (Figure 1e). While an increase in pocket volume from 2 mL to 10 mL only decreased the maximum velocity magnitude in peristalsis by <1%, a similar volume increase for segmental motility led to a substantial increase (77%). This is because in segmental motility, at a constant period, the epithelial surface covers a larger radial distance as pocket size increases, thereby generating greater momentum and higher velocity values. Consequently, the circulation regions induced in larger pockets tended to be more skewed. In both segmentation and peristalsis, higher shear stress was observed near the epithelium and in the most occluded regions.

The velocity and shear-stress magnitudes predicted in our CFD simulations are consistent with values reported for small-intestinal hydrodynamics. Human duodenal peristalsis typically produces flow velocities of 0.17–0.33 mm/s in fasting conditions and up to 5–20 mm/s during phase III of the MMC (Vantrappen et al. 1981; Palmada et al. 2022; Hasan 2024; Naranjani 2025), with comparable flow fields observed in animal studies (Jeffrey et al. 2003). Direct measurement of epithelial shear stress *in vivo* is not feasible; commonly cited physiological values (≈0.2–8 mPa) originate primarily from *in vitro* systems such as gut-on-chip platforms where flow rates and peristaltic deformation are tightly controlled (Jung Kim et al. 2012; Lindner et al. 2021; Fois et al. 2021). In contrast, CFD enables full spatial resolution of near-wall hydrodynamics under physiologically motivated segmentation and peristalsis patterns.

Across all simulated conditions, the median surface-averaged shear stresses were 5.0 mPa, 9.9 mPa, and 14.9 mPa, depending on motility and fluid types (Figure 1c). The first two values (5.0 and 9.9 mPa) fall within or slightly above the commonly reported 0.2–8 mPa range for *in vitro* intestine models, while the highest median value (14.9 mPa) arises specifically under peristalsis with the nutritional drink. This elevated shear is expected, as peristalsis generates more forceful contractions and produces higher near-wall stresses, consistent with the shear increases observed during stronger motility events such as MMC phase III. Importantly, these values represent surface-averaged shear stress along the epithelial wall; localised higher shear pockets appear only under extreme ORs and remain confined to small regions rather than characterising the global near-wall environment. Consistent with experimental and computational literature, our simulations also produced maximum velocity magnitudes between approximately 0.12 cm/s and 3.32 cm/s (Figure 1a).

Consistent with these physiological considerations, we next quantified how shear stress behaves under the different simulated conditions. Shear stress influences both the dissolution of small molecules and the mechanical stability of macromolecular drugs, which can be sensitive to mechanical forces (Moino et al. 2024). Therefore, we determined the average and maximum shear stress at the epithelial surface (Figure 1c). A multifactor analysis was conducted to investigate how these metrics changed when motility intensity, pocket contractility, pocket size, motility type, and intestinal fluid were varied. We observed that replicating the fed state of the intestinal fluid with a nutritional drink, a non-Newtonian fluid, resulted in a 135% increase in average shear stress and a 175% increase in maximum shear stress, although the distribution patterns remained similar. In the segments exposed to the nutritional drink, the most notable deviations in maximum shear stress occurred with more intense motor activities, specifically in the 10 mL pockets (Appendix B). Considering the different variability factors and their respective ranges, peristalsis significantly increased both the average and maximum shear stress at the epithelial surface.

To further understand how variations in motility intensity, fluid composition, contractility, and pocket size influence the transient flow structures during segmentation, we conducted a pairwise comparison across four time points within a single segmentation cycle (Figure 1b and Movie S1). In particular, we highlight the phenomena of separation and amalgamation of adjacent water pockets at time points $t={0.25t}_{p}$ and $t={0.75t}_{p}$ . The interface between the two counter-rotating circulation regions undergoes changes in surface area (through contraction or expansion). This change caused a radial flow to emerge at the corresponding axial position along the centreline, moving either inward or outward to conserve mass. The direction of this radial flow shifts with each alternation between the expansion and contraction. Subsequently, the circulation zone diminishes, paving the way for the formation of new zones.

### 3.2. Concentration transport

#### 3.2.1. Radial flow enhances surface concentrations of insulin and C10

**Figure 2.**
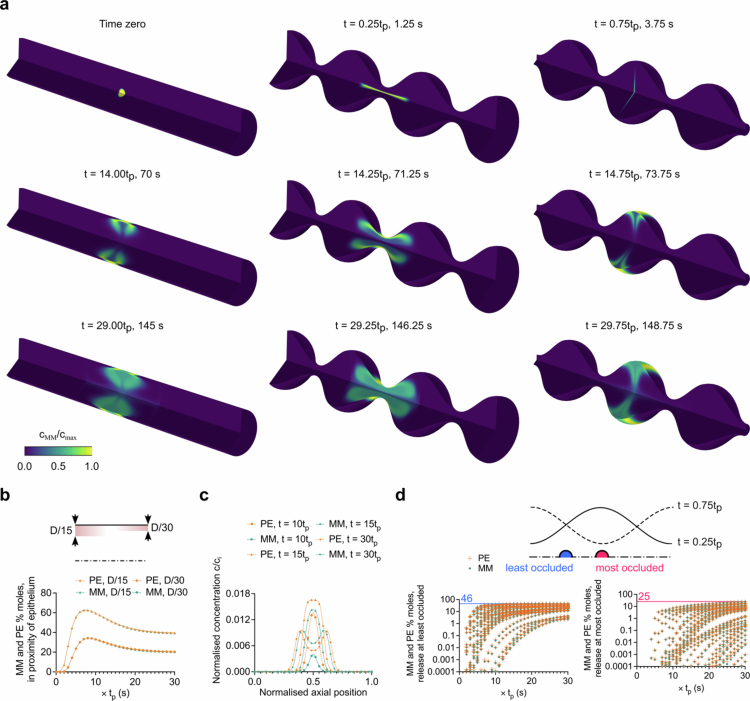
Intraluminal transport dynamics of insulin and C10 during segmental motility with moderate intensity (*t*_*p*_ = 5 s) and nutritional drink as the intestinal fluid**. a** Release and subsequent transport of dissolved insulin during the course of thirty periods. The contours show the evolution of normalised concentrations through stretching and thickening phenomena induced by recursive contractions and expansions. **b** Comparative analysis of radial transport towards the epithelial surface. The plot depicts the percentage of released insulin and C10 moles within a thin layer (thicknesses of D/15 and D/30 based on approximations of villi length) adjacent to the epithelial surface as a function of time. **c** Normalised concentration profiles of insulin and C10 at different time intervals. The plot reveals the spatial distribution and temporal evolution of drug concentrations over the epithelial surface pertaining to the release pocket. **d** Percentage of insulin and C10 moles that escape the release pocket for release at the least and most occluded positions as a function of time. The dot plots quantify localised mechanisms of segmental motility.

Next, we focused on the segmentation-driven intraluminal transport of insulin and C10. Our simulated segmentation induced a repetitive pattern of stretching and compression of the dissolved drug particle in both axial and radial dimensions (Figure 2a and Movie S2). This process coincided with the sequential emergence of counter-rotating vortices. The flow structures generated by segmentation facilitated time-dependent fluid transport in opposing directions at the pocket centre, affecting both axial and radial movements. However, the momentum magnitudes of these movements differed significantly in the axial and radial directions (Figure 1b). Notably, the momentum of the radial flow was greater when moving towards the epithelial surface during pocket expansion than when directed towards the axial line during contraction. This differential momentum led to a gradual shift in drug concentration from its point of release on the axial line towards the epithelial surface, underscoring the dominance of radial transport due to segmentation. This mechanism contrasts with peristaltic movement, in which axial fluid transport prevails along the wave propagation (Naranjani et al. 2023). The mole percentage of MM and PE escaping the pocket for release at the pocket centre (the most occluded position) was < 25%, whereas it was < 46% at one-quarter of a wavelength away from the centre (the least occluded position).

We quantified the molar percentages (relative to the total released amount) near the epithelial surface to elucidate the dynamics and eventual fate of the drug molecules near the epithelial surface. Our methodology involved analysing the transport of drug molecules from the bulk to thin layers with thicknesses of one-fifteenth and one-thirtieth of the intestinal pocket diameter, roughly corresponding to the length of the intestinal villi (Lim et al. 2015). We discovered that the molar percentages of both insulin and C10 in the thicker and thinner layers peaked at 62% and 32%, respectively, after seven and eight periods (Figure 2b). Following an initial surge, the percentages decreased at a rate that slowed over time. Consequently, the movement towards the epithelial surface did not lead to a steady build-up. Rather, the ongoing segmentation contributed to intraluminal mixing, resulting in a reduction in the mole percentages to 39% and 19% for the respective layers after 30 periods (Figure 2b). The molecular diffusivities of C10 and insulin differed by a factor of 3.15 for 100 mM C10 and 5.50 for 1 mM C10. Despite this, the amount at the surface was similar for the two compounds, indicating a convection-dominated mass transfer mechanism (Naranjani et al. 2023).

The simulations further revealed that detectable concentration levels ( $c/c_{i}>0.001$ ) at the epithelial surface became evident after five cycles, displaying a bell-shaped distribution that intensified over time (Figure 2c). Initially a single-peaked bell shape, this distribution gradually evolved and split into two distinct bell-shaped curves. Although differences in the amounts of insulin and C10 near the epithelial surface were small, the concentration profiles diverged significantly. Insulin exhibited delayed onset and peaked at a concentration 16% lower than that of C10. This discrepancy stems from the minimal relative velocity of the intestinal fluid to the epithelial surface in the direction normal to the surface within the boundary layer, rendering diffusion the predominant mode of transport. Consequently, the enhancer with its lower molecular diffusivity tends to accumulate more rapidly and achieve a higher maximum concentration.

#### 3.2.2. Light segmentation intensity maximises surface concentrations of insulin and C10

The intensity of segmental motility is determined by the duration of periodic movements executed by the intestinal wall. Our previous study, along with other findings, suggests that slower motility enhances the accumulation of MMs at the epithelial surface (Naranjani et al. 2023). Kimura et al. demonstrated that prolonged residence time of poly(vinyl alcohol)-gel spheres (PVA-GS) containing insulin and protease inhibitors in the ileum resulted in increased insulin bioavailability (Kimura et al. 1996). This indicates that prolonged retention plays a crucial role in enhancing absorption. Additionally, Morishita et al. showed that a longer residence time of an absorption enhancer (sodium decanoate) together with a poorly absorbed drug (sodium cefazolin) led to improved drug uptake compared to shorter residence times (Baluom et al. 1998). These findings support our hypothesis that slower wave speed in the duodenum may improve drug accumulation at the epithelial surface.

**Figure 3.**
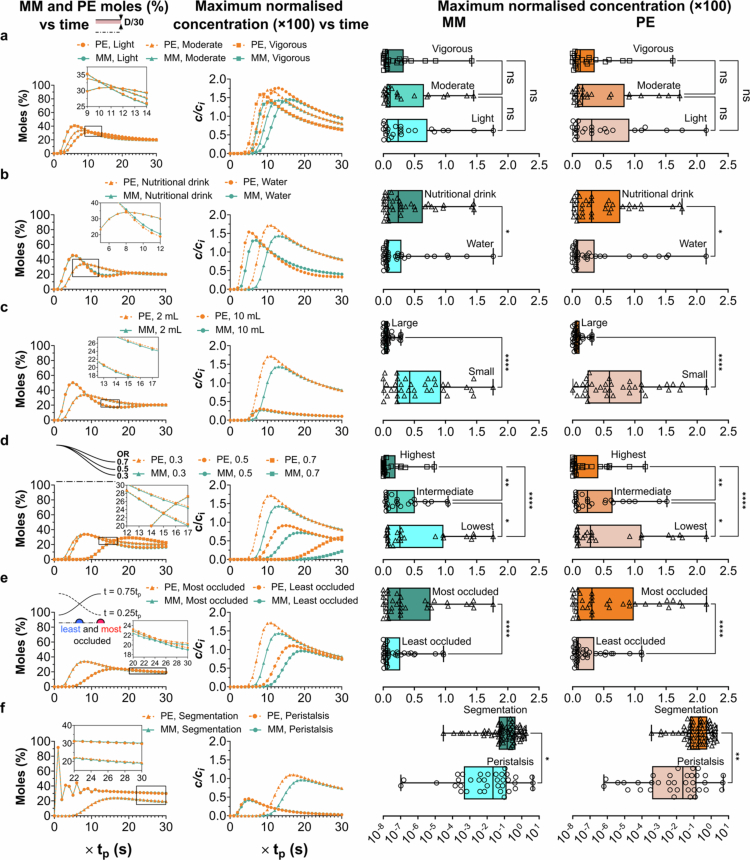
Impact of intestinal variability on intraluminal transport of insulin and C10, relative to the baseline segmentation condition (moderate intensity, nutritional drink, 2 mL pocket size, OR of 0.3, and release at most occluded position). Each panel shows the percentage of drug molecules in the proximity of the epithelium and the maximum concentration at the corresponding surface as a function of time. Multifactor analyses of both compounds for the maximum concentrations were included while considering all cases for each class. **a** Assessment of variations in segmentation intensity (light, moderate, and vigorous, with *t*_*p*_ = 7, 5, and 3 s, respectively). **b** Comparison of representative luminal fluids (water versus nutritional drink). **c** Effects of pocket size (2 and 10 mL). **d** Effects of pocket contractility (OR 0.3, 0.5, and 0.7). **e** Comparative assessment of release under two extremely different conditions (the least and most occluded positions). **f** Head-to-head comparison of segmentation and peristalsis motilities. Statistical analyses were performed using Dunn's multiple comparisons test, Mann-Whitney U test, or Kruskal-Wallis test, as appropriate, based on the results of the normality testing (See Statistical analysis section in Methods). Significance levels are indicated by asterisks, where *, **, ***, and **** denote *p*-values of < 0.05, < 0.01, < 0.001, and < 0.0001, respectively.

Therefore, we aimed to assess the influence of the segmentation intensity on intraluminal transport. The movement period was categorised into 3 s (vigorous), 5 s (moderate), and 7 s (light) to represent biologically relevant cyclic motions (Du et al. 2010). We found that higher motility intensities resulted in a larger fraction of released molecules near the epithelial surface, within a thin volume of D/30 thickness. Surprisingly, this did not translate into increased surface concentrations (Figure 3a, Appendix C). Rapid mass transfer associated with more vigorous motility led to earlier peaks in mole percentage near the epithelium. These occurred at six, eight, and 11 periods (equivalent to 18, 40, and 77 s) post-release for the vigorous, moderate, and light motility intensities, respectively. This suggests that increased convection levels diminish the time available for drug molecules near the epithelial surface to undergo diffusion towards it, thus hindering the attainment of higher concentration levels (Figures 2c and 3a). Notably, the segmentation motility of light intensity ultimately resulted in maximum concentration levels at the epithelial surface, which were 5% higher for insulin and 10% higher for C10 than those achieved with vigorous segmentation.

Conversely, a relatively quicker ascent to the peak mole percentage was followed by a slower decline (Figure 3a). However, there were moments when the values for less intense segmentation surpassed those for higher intensity, eventually leading to a higher maximum. This pattern was parallel to that of the maximum normalised concentration data. Not only does lighter intensity segmentation result in higher peaks, but these elevated values are also maintained until the conclusion of the simulation. Specifically, at the termination time point, light-intensity segmentation increased the insulin concentration values by 47% and 46% for C10 compared with vigorous segmentation.

To extend our analysis and incorporate potential sources of variability, we conducted a multifactor analysis (box plots in Figure 3a). This involved categorising three levels of intensity and including all simulated cases for each of the following scenarios: i) different pocket sizes (2 mL and 10 mL); ii) contraction level (OR of 0.3, 0.5, and 0.7); iii) release location (least and most contracted axial positions); and iv) intestinal fluid composition (water versus nutritional drink). Our initial model findings used a 2 mL pocket, an OR of 0.3, a nutritional drink as the fluid, and release at the pocket centre. The multifactor analysis corroborated the findings of our base model, indicating that light motility culminates in higher concentrations. We compared the average value of the maximum concentrations for all cases in each category and found that the moderate and light intensities for insulin were 33% and 200% higher, respectively, than the vigorous intensity (Figure 3a). For C10, the increase was 26% for moderate intensity and 146% for light intensity.

#### 3.2.3. Viscous fluids enhance surface concentrations of insulin and C10

Given the variability in an individual’s diet, the composition of the intestinal fluid is inherently dynamic, leading to changes in the rheological properties of the chyme within the small intestine. To assess the influence of intestinal fluid viscosity on the luminal transport of MMs and PEs, we used two distinct media: water and a nutritional drink (MiniMax, diluted with 10% water). Water mimicked a diluted environment in the small intestine, and the nutritional drink was viscous and nutrient-dense. Rheological tests were conducted to determine the density and apparent viscosity of the nutritional drink (Appendix D). These tests revealed shear-thinning behaviour, which we modelled using a Power Law formula.

To accurately reflect the effects of viscosity and temperature on molecular diffusion, we adjusted the diffusivity values of the molecules using the Stokes-Einstein equation. Prior to adjustment, the diffusion coefficient of insulin was noted as 1.11 × 10^−6^ cm^2^/s, and the concentration-dependent molecular diffusivity of C10 was taken from our previous investigations (Patil et al. 2017; Naranjani et al. 2023).

After establishing the modelling input parameters, we conducted numerical simulations for our baseline scenario, using water and a nutritional drink (Figure 3b). These simulations showed that water led to a higher peak mole percentage near the epithelial surface than that of the nutritional drink. For the more viscous fluid, the peak mole percentage of insulin was delayed by $3t_{p}$ , and the normalised concentration peak was delayed by $6t_{p}$ . This delay underscores the significant influence of fluid viscosity on insulin transport dynamics near the epithelial surface.

The peak flow velocity of the nutritional drink exceeded that of water by 19%. This could lead to the incorrect conclusion that the concentration levels at the epithelial surface were lower owing to enhanced convection rates (Figure 2b). However, the increased velocity was confined primarily to the vicinity of the pocket centre, and notably, the flow velocity near the epithelial surface was reduced for the nutritional drink. The higher viscosity of the nutritional drink, which restricts fluid mobility, results in less pronounced vortices whose centres are positioned further from the epithelial surface. Consequently, the diminished momentum and shear levels near the epithelial surface effectively elevated the concentration levels by extending the residence time in proximity of epithelium. Our multifactor analysis revealed that, on average, the peak concentrations at the epithelial surface were 227% for insulin and 258% for C10, a substantial increase compared to those for water.

#### 3.2.4. Smaller intestinal pockets drastically increase surface concentrations of insulin and C10

Intestinal pocket sizes fluctuate both intra- and inter-individually. Therefore, we explored how these size variations influence the concentration levels at the epithelial surface during segmentation. The number of moles near the epithelium for different pocket sizes was reminiscent of those observed with variations in the intestinal fluid viscosity. This suggests that pocket size could exert a similar effect on drug transport dynamics, but the impact on surface concentration levels was markedly distinct. Specifically, reducing the pocket size fivefold from 10 mL to 2 mL under segmentation dramatically increased the maximum concentration levels of insulin by 424% and C10 by 469% (Figure 3c).

The epithelial surface in the 10 mL pocket is required to cover a radial distance approximately 1.7 times greater than in the 2 mL pocket within the same period. This necessitates the maintenance of a higher momentum at the epithelial surface, leading to enhanced convection and mixing within the pocket (Figure 1b,e). Contrary to the effects associated with intestinal fluid viscosity, for the larger pocket volume, areas of higher velocity extended beyond the pocket centre to include regions close to the epithelial surface (Figure 1b). This condition promotes increased convection near the epithelium, which impedes the process of molecular accumulation at the surface. The average maximum concentrations of insulin and C10 in 2 mL pockets were more than 6- and 8-fold higher than in 10 mL pockets, respectively. This demonstrates the significant impact of pocket size variability on the concentration levels at the epithelial surface for identical doses.

#### 3.2.5. Reduced pocket contractility weakens transport toward the epithelium

Contractile dysfunction within the small intestine can arise from a variety of factors, including neurodegenerative disorders (Coletto et al. 2019), redox imbalances (de Souza et al. 2018; Karatzaferi et al. 2019), and deficiency in probiotics (Dimidi et al. 2017). The inability of the intestine to sustain regular contractions presents a significant risk to intestinal transit times, affecting the luminal flow patterns and the transport of molecular drugs and nutrients from the bulk fluid to the epithelial surface. To evaluate the influence of pocket contractility on drug transport, we analysed three levels of contraction, characterised by ORs of 0.3, 0.5, and 0.7. These ratios were derived from the ratio of the minimum to average radius of the intestinal epithelium (Figure 1a). Compared with the highest contraction level (OR = 0.3), the lower contraction level (OR = 0.7) reduced the uptake of insulin and C10 molecules by 38% from the pocket centre to a layer (D/30 thickness) adjacent to the epithelial surface. This phenomenon can be attributed to two main factors: i) a decrease in maximum luminal velocity magnitude for OR = 0.7 (Figure 1b); and ii) the alteration of vortical structures, both of which diminish radial transport towards the epithelial surface. Our multifactor analysis further revealed that the average value of maximum concentrations decreased substantially for insulin (73%) and C10 (71%) in the group with the lowest contractility compared with the group with the highest contractility (Figure 3d).

#### 3.2.6. Release at more occluded positions boosts transport to the epithelium

For the release location, we evaluated two distinct scenarios: i) release at the pocket centre, where epithelial radial contraction is maximised (most occluded position); and ii) release at a distance λ/4 away from the pocket centre along the centreline, where radial contraction is minimal (i.e. the least occluding position, Figure 2d). Under segmentation the release of the drug molecules at the pocket centre, where contraction intensity is greatest, resulted in a greater translocation of molecules towards the epithelium. The accumulation of molecules at this location occurred sooner than when released off-centre, leading to elevated concentrations at the epithelial surface. This phenomenon is attributed to the enhanced radial flow towards the epithelium in areas experiencing significant epithelial displacement, such as the pocket centre and the intersections between pockets (Figure 1b).

Conversely, the cyclic nature of segmental expansions and contractions causes the luminal flow to oscillate in opposite directions at any given point. Thus, releasing molecules along the centreline in areas of lesser radial contraction leads to alternating axial transport until the molecules reach areas where the epithelial layer experiences greater radial displacement. Subsequently, radial transport towards the epithelial surface alternated similarly (Figure 2a and Movie S2). Therefore, releasing drugs in regions of high occlusion (characterised by more intense radial transport) facilitates more effective intraluminal transport of drugs to the epithelial surface.

#### 3.2.7. Segmentation outperforms peristalsis in enhancing drug transport to the epithelium

After exploring segmentation in the context of luminal transport mechanisms for biologics with PEs, we compared the intraluminal transport and arrival timings of insulin and C10 using peristalsis as the motor function. Peristalsis facilitated axial transport, with molecules moving along the wave path, whereas segmentation involved more radial transport (Figure 2d). Consequently, unlike segmentation, the peak concentration levels at the epithelial surface in peristalsis were not found at the release site, but further downstream across several pocket wavelengths (Naranjani et al. 2023).

To model a realistic intestinal segment undergoing peristalsis, we calculated that the simulation domain must be composed of 30 pockets in length and the simulation run over 30 periods. This ensures that the drug molecules stay within the computational domain during the course of simulation. Initial post-release observations under peristalsis revealed fluctuations in drug molecule translocation to the epithelial vicinity (Figure 3f). Notably, after one period post-release, over 90% of the molecules were within a distance of less than one-thirtieth of the intestinal pocket diameter from the epithelium. However, one period later, fewer than 30% of the molecules remained at that distance from the epithelial surface. This was due to the formation of a ‘trapped zone’ in peristalsis, in which drug molecules circulated within vortices (Movie S2). This pattern was then noticeably repeated for at least 10 periods (Figure 3f). Segmentation resulted in maximum normalised concentrations of insulin and C10 that were 128% and 137% higher than those under moderate peristalsis with a nutritional drink. This efficiency in segmental motility stems from enhanced molecular diffusion via localised transport processes and translocation of high-concentration drug regions to the vicinity of the epithelial surface.

Our multifactor analysis considered variabilities, such as motility intensity, pocket volume, release location, and contraction level. The average maximum normalised concentration for both insulin and C10 with segmentation significantly exceeded that of peristalsis by factors of 6.7 and 8.0, respectively. This underscores the effectiveness of segmentation in promoting efficient intraluminal drug transport to the epithelial surface and presents substantial implications for the design of oral biologic delivery strategies.

##### 3.3. Colocalisation score (CS) and machine learning insights

After quantifying the intraluminal mass transfer of our model MM and PE from the intestinal bulk to the epithelial surface, we proposed a metric to score their co-delivery. We define the CS as:

(4) $$\mathrm{CS}=\frac{{\overset{®}{C}}_{\mathrm{MM}}{\overset{®}{C}}_{\mathrm{PE}}}{1+\sqrt{{\overset{®}{\mathrm{dt}}}^{2}+{\overset{®}{\mathrm{dz}}}^{2}+{\overset{®}{\mathrm{dr}}}^{2}}}$$

where ${\overset{®}{C}}_{\mathrm{MM}}$ and ${\overset{®}{C}}_{\mathrm{PE}}$ are the maximum concentrations of MM and PE at the epithelial surface (in time for each case) which are normalised based on the maximum concentration values among all considered cases. The denominator accounts for the corresponding temporal and spatial differences between the maximum values of MM and PE arrival time at the epithelium for each case. The CS score takes any value between 0 and 1, with larger values monotonically showing higher degrees of colocalisation.

###### 3.3.1. What factors most influence macromolecule and enhancer colocalisation?

**Figure 4.**
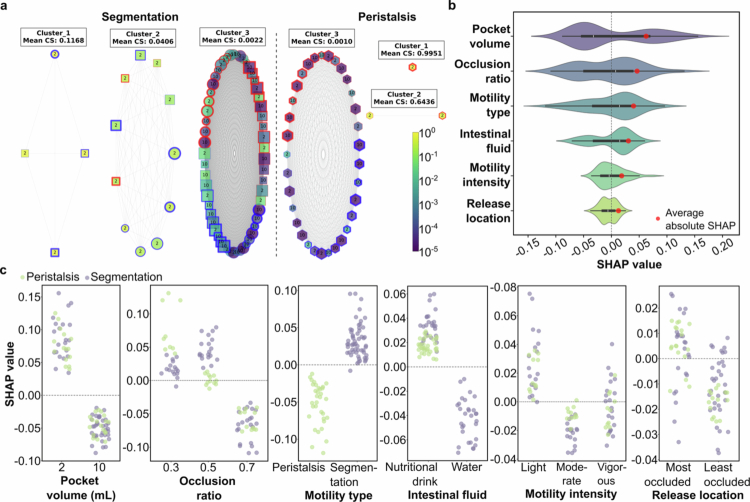
Clustering and SHapley Additive exPlanations (SHAP) analysis of the CS. **a** K-means clustering of simulated cases based on CS. Each case is represented as a node; the size of the node depicts the contractility level, with smaller sizes indicating greater contractions. In the context of segmentation, water and nutritional drink are represented by circles and squares, respectively, while peristaltic motility is represented by hexagons. The border colour of nodes represents the initial release location, with blue indicating release at the pocket centre and red indicating release off-centre. Motility intensity is depicted by the thickness of the node border, from the thinnest (most vigorous) to the thickest (least intense). **b** SHAP value distribution for each feature in the XGBoost model, sorted by mean absolute SHAP value. The violin plots display the distribution of SHAP values for each feature, with red dots indicating the average absolute SHAP value. Features are ordered from top to bottom based on their impact on the model, with pocket volume, pocket contractility, and motility type being the most significant. **c** SHAP dependence plots for all the variability factors: pocket volume, pocket contractility, motility type, intestinal fluid, intensity, and release location. SHAP dependence plots display one SHAP value per simulated case and therefore do not include error bars. A single y-axis label (‘SHAP value’) is used for all subplots to maintain consistency across the panel.

We used K-means clustering to group all the simulated cases based on CS (Figure 4a). For segmentation, the two clusters with the highest CS were enriched with cases involving the nutritional drink. This implies that they have a greater level of efficacy for the intraluminal transfer of MMs and PEs to the epithelial surface (Figure 4a). Overall, segmentation outperformed peristalsis in terms of the CS metric. The mean CS score for the third cluster in the segmentation was more than two-fold greater than that of peristalsis. This is despite the segmentation cluster containing the low-performing fluid (water).

To further investigate the impact of variability factors on the CS within the largest cluster (Cluster 3), which contained the majority of data points, we employed an XGBoost regression model. The final model demonstrated improved predictive performance which achieved mean squared error (MSE) of 0.0336, a root mean squared error (RMSE) of 0.1834, and an R-squared (R²) value of 0.1052 on the test set. The relatively low R² value reflects the limited size of the dataset and the fact that the model receives only coarse scalar descriptors of the CFD cases. Because the CS arises from highly nonlinear flow behaviour captured in the full velocity and concentration fields, several fine-scale hydrodynamic features that influence CS cannot be encoded without additional feature engineering. Nonetheless, the model is sufficient for feature attribution analysis, and the SHAP outputs reliably identify which variability factors contribute most strongly to CS.

We used SHapley Additive exPlanations (SHAP) analysis to identify the most influential factors affecting the CS. The SHAP analysis revealed that pocket volume, pocket contractility, and motility type were the top three most impactful factors on the CS (Figure 4b). The violin plots in Figure 4b display the distribution of SHAP values for each feature, with larger mean absolute SHAP values indicating greater overall importance to the model's predictions. To understand how different values of each variability factor influenced the CS, we examined the SHAP dependence plots (Figure 4c). These plots illustrate the relationship between the feature values and their corresponding SHAP values, showing whether specific variations in the factors have a positive or negative effect on the CS.

For instance, cases with a pocket volume of 2 mL had SHAP values predominantly above zero, reflecting a positive impact on the CS, while larger volumes (10 mL) had SHAP values below zero. Similarly, an OR of 0.3 showed higher SHAP values compared to higher ORs, indicating that greater contractility positively contributes to the CS. Segmentation motility had higher SHAP values than peristalsis, demonstrating a more substantial positive effect on the CS. These findings confirm that pocket volume, pocket contractility, and motility type are critical factors in achieving optimal colocalisation of MMs and PEs at the epithelial surface.

## 4. Discussion and perspectives

Our study demonstrated how intestinal motility dynamics, fluid composition, pocket contractility, and release location influence the transport of MMs and PEs within the intestinal lumen. We found that segmentation motility significantly enhanced the concentration of MMs and PEs at the epithelial surface compared to peristalsis. Specifically, segmentation increased the average maximum normalised concentrations of insulin by 128% and C10 by 137% compared to moderate-intensity peristalsis in a high-calorie nutritional drink. Our multifactor analysis revealed that segmentation achieved 6.7-fold and 8.0-fold higher average maximum normalised concentrations of insulin and C10, respectively, compared to peristalsis. This indicates that segmentation is more effective in promoting the localised transport of dissolved molecules to the epithelium.

Moreover, we found that light-intensity segmentation maximised surface concentrations of insulin and C10. Compared with vigorous segmentation, light-intensity segmentation increased insulin and C10 concentrations at the epithelial surface by 47% and 46%, respectively. This suggests that allowing sufficient time for molecular diffusion during slower segmentation cycles enhances the accumulation of MMs and PEs at the epithelium.

The fluid composition also played a significant role. Using a viscous nutritional drink as the intestinal fluid, we observed a substantial increase in the peak concentrations at the epithelial surface compared to water, with increases of 227% for insulin and 258% for C10. The higher viscosity reduced fluid mobility near the epithelial surface, extending the residence time and facilitating greater accumulation of molecules. Additionally, smaller intestinal pocket sizes drastically increased surface concentrations. Reducing the pocket volume from 10 mL to 2 mL led to a 424% increase in the maximum concentration of insulin and a 469% increase for C10. This effect is attributed to reduced convection and mixing in smaller pockets, allowing more time for molecules to diffuse to the epithelium.

Higher pocket contractility (lower OR) also enhanced transport to the epithelium. An OR of 0.3 (most contracted) increased the average maximum concentrations of insulin and C10 by 73% and 71%, respectively, compared to an OR of 0.7 (least contracted). The release location influenced the transport efficacy as well. Releasing molecules at the most occluded position resulted in greater translocation towards the epithelium compared to release at less occluded positions.

To quantify the co-delivery efficacy of MMs and PEs to the epithelial surface, we developed a CS, which accounts for both the maximum concentrations and the temporal and spatial alignment of their peaks. Using this metric, we observed that segmentation motility, particularly when involving the nutritional drink, achieved significantly higher CS values than peristalsis. This indicates superior co-delivery of MMs and PEs under segmentation motility conditions.

Further analysis using an XGBoost regression model revealed that pocket volume, pocket contractility, and motility type were the most influential factors affecting the CS. Specifically, smaller pocket volumes (2 mL), higher contractility (lower ORs), and segmentation motility positively contributed to higher CS values. These findings confirm the critical role of these factors in achieving optimal colocalisation of MMs and PEs at the epithelial surface.

While the model captured several consistent patterns influencing the CS, the R² value of 0.1052 reflects the limited explanatory power of the six variability factors used as inputs to the XGBoost model. This outcome is expected for two reasons. First, the dataset is relatively small, and the test set on which R² is computed represents only a fraction of the available simulations, making the evaluation sensitive to sampling variability. Second, the CS arises from highly nonlinear hydrodynamic interactions captured in the full CFD flow fields, whereas the machine-learning model receives only coarse scalar descriptors. Many fine-scale structures such as vortex strength, spatial asymmetries, and transient radial jets contribute to CS but cannot be encoded without dedicated CFD-based feature engineering. It is worth noting that our goal was to identify which variability factors most strongly influence colocalisation rather than to build a predictive surrogate. Accordingly, the SHAP-based feature attribution remains informative and valid even when absolute predictive accuracy is modest.

In this study, we imposed a zero-absorption condition at the epithelial wall to isolate the hydrodynamic mechanisms governing colocalisation. Although appropriate for analysing transport to the surface, real intestinal physiology involves transcellular and paracellular uptake, enzymatic degradation, and dynamic mucus turnover, all of which reduce luminal concentrations and may alter both the magnitude and timing of peak values at the epithelium. Furthermore, microstructural features such as villi and spatial variability in mucus thickness can modulate near-wall velocities and convection patterns, potentially affecting the extent to which segmentation-driven radial transport enhances surface concentrations. These represent important avenues for future model refinement. Future validation steps may also include comparing the timing and magnitude of predicted epithelial concentrations with pharmacokinetic data from oral insulin studies once absorption mechanisms are incorporated into the model. Finally, our simulations assumed perfectly periodic motility patterns for segmentation and peristalsis to enable a controlled mechanistic comparison. *In vivo*, intestinal motility exhibits cycle-to-cycle irregularity, which may shift the spatial or temporal location of maximum concentrations. Incorporating such physiological variability in future simulations will further improve translational relevance.

Although our results indicate that fed-state segmentation provides hydrodynamic conditions that enhance the localised delivery of MMs and PEs, current oral biologic development often favours fasted-state dosing for practical reasons. The fasted state reduces inter-individual variability in gastric emptying, luminal viscosity, and pH, which simplifies dose–exposure relationships during clinical evaluation. This is relevant for gastric-targeted systems such as oral semaglutide with SNAC, which rely on stabilising a narrow gastric absorption window rather than promoting small-intestinal transport. In contrast, formulations intended for small-intestinal absorption may benefit from the enhanced mixing and near-wall transport associated with fed-state segmentation, yet exploiting these conditions *in vivo* remains challenging because fed-state motility is more heterogeneous and patient-specific. Our model therefore provides insight into the hydrodynamic potential of segmentation while highlighting why fed-state delivery is underused in practice.

Our findings underscore the importance of segmentation motility and specific physiological conditions in maximising the intraluminal transport and co-delivery of MMs and PEs to the epithelial surface. By optimising factors such as motility intensity, fluid viscosity, pocket size, contractility, and release location, it may be possible to enhance the bioavailability of orally administered MMs and improve the efficacy of PEs. These insights can inform the design of oral drug delivery strategies that leverage the dynamic mechanical processes of gastrointestinal physiology. For example, formulations or excipients that modulate intestinal motility toward segmentation-like patterns or increase luminal viscosity may enhance the localised concentration of therapeutics at the absorption site.

Our computational models provide a framework for predicting how variations in physiological conditions affect drug transport within the intestine, including the inter-individual differences seen in conditions such as IBS or diabetes. By simulating the intraluminal transport of larger MMs and smaller molecules like PEs based on a person's unique motility patterns, fluid composition, and pocket geometry, the model can quantify how these differences shift exposure at the epithelial surface and thereby guide potential adjustments in dosing or release characteristics. Tailoring oral drug delivery strategies to individual patients has the potential to enhance the bioavailability of therapeutics and improve treatment efficacy, which paves the way for more effective and personalised medical interventions.

## Supplementary Material

Supplementary material Supplementary material_Revised.docx

## Data Availability

The data are made available upon request.

## Code availability

The COMSOL simulation files used in this study are available in a GitHub repository: https://github.com/benyamin-naranjani/intestinal-motility-modelling.git.
