# Supplementary material for: Mechanics of small intestine motility for oral macromolecular delivery: modelling segmentation versus peristalsis
Source: Drug Deliv. 2025 Dec 24;33(1):2607779. doi: 10.1080/10717544.2025.2607779 (PMC12777773; doi:10.1080/10717544.2025.2607779)

# Appendices and supplementary material Mechanics of small intestine motility for oral macromolecular delivery: Modelling segmentation versus peristalsis

Benyamin Naranjani1,2, Shakhawath Hossain1, Marco Tjakra1,2, Pardis Azhand1, Christel Bergström1,2, Patrick Sinko1, Per Larsson1,2

1Department of Pharmacy, Uppsala Biomedical Center, Uppsala University, 751 23 Uppsala, Sweden

2Swedish Drug Delivery Center (SweDeliver), Uppsala University, 751 23 Uppsala, Sweden

## Appendix A – Modelling a time-dependent velocity distribution at the intestinal wall boundary to mimic intestinal segmentation

In the study of segmentation, periodicity in both time and space is crucial for accurately modelling the underlying biological processes. To address these periodic characteristics, we proposed a radial velocity function for the epithelial surface. This function is formulated by multiplying two cosine functions, one representing the spatial periodicity in the axial direction and the other representing the temporal periodicity. This approach allowed us to incorporate key parameters, such as wavelength and time period, directly into the model. We propose the velocity function to have the following form:

| () | $U_{r}\left( Z,t \right)=2\pi\frac{\delta}{t_{p}}cos(2\pi\frac{Z}{\lambda})cos(2\pi\frac{t}{t_{p}})$ | (S1) |
| --- | --- | --- |

where $\lambda$ is the wavelength corresponding to one water pocket and $t_{p}$ is the time required for the wave to undergo one cycle. This velocity function successfully models the periodic characteristics of segmental motility; however, it introduces an unphysical phenomenon of fluid suction and ejection when incorporated into the CFD model. Such behaviour is not biologically plausible and can lead to significant errors in the simulation and analysis. To rectify this issue, we introduced a correction term to the velocity function as follows:

| ) | $U_{r}\left( Z,t \right)=2\pi\frac{\delta}{t_{p}}cos\left( 2\pi\frac{Z}{\lambda} \right)cos\left( 2\pi\frac{t}{t_{p}} \right)+f\left( t \right)$ | (S2) |
| --- | --- | --- |

The correction term was intended to ensure volume conservation within the system. The corrected velocity function not only adheres to the observed periodicity, but also respects the fundamental physical laws (such as the conservation of mass and volume). To determine the correction term, we imposed a mathematical constraint to set the potential variations in volume equal to zero as follows:

| ) | $dv=\int_{0}^{\lambda} 2\pi G\left( Z,t \right)U_{r}\left( Z,t \right)dtdz=0$ | (S3) |
| --- | --- | --- |

where $G\left( Z,t \right)$ denotes the physical position of the epithelial boundary. We obtained this by integrating (Eq. S2), resulting in:

| () | $G\left( Z,t \right)=\delta cos\left( 2\pi\frac{Z}{\lambda} \right)cos\left( 2\pi\frac{t}{t_{p}} \right)+r_{0}+\int f\left( t \right)dt$ | (S4) |
| --- | --- | --- |

where $r_{0}$ is the radius of boundary position when the contraction is uniform across the epithelial surface. Substituting Eqs. S2 and S4 into Eq. S3 results in the following ordinary differential equation (ODE):

| () | $f\left( t \right)\int f\left( t \right)dt+r_{0}f\left( t \right)+\frac{\pi\delta^{2}}{{2t}_{p}}sin\left( 4\pi\frac{t}{t_{p}} \right)=0$ | (S5) |
| --- | --- | --- |

Solving the above ODE gives us the radial velocity correction term:

| () | $f\left( t \right)=\frac{-\frac{\pi\delta^{2}}{t_{p}}sin\left( 4\pi\frac{t}{t_{p}} \right)}{2\sqrt{r_{0}^{2}+\frac{\delta^{2}}{4}\left( cos\left( 4\pi\frac{t}{t_{p}} \right)-1 \right)}}$ | (S6) |
| --- | --- | --- |

## Appendix B – Shear stress distributions for segmentation

Here, we provide complementary information to Figure 1a, detailing the shear stress distribution in the segmentation cases. Specifically, we compared the nutritional drink and water at different time points. Shear stress was significantly greater for the nutritional drink than for water in the lumen at the pocket centre, pocket intersections, and at locations with minimum contractility over the epithelial surface (Figure S1c). The relatively larger values of dynamic viscosity are the prominent cause yielding higher shear stress levels.


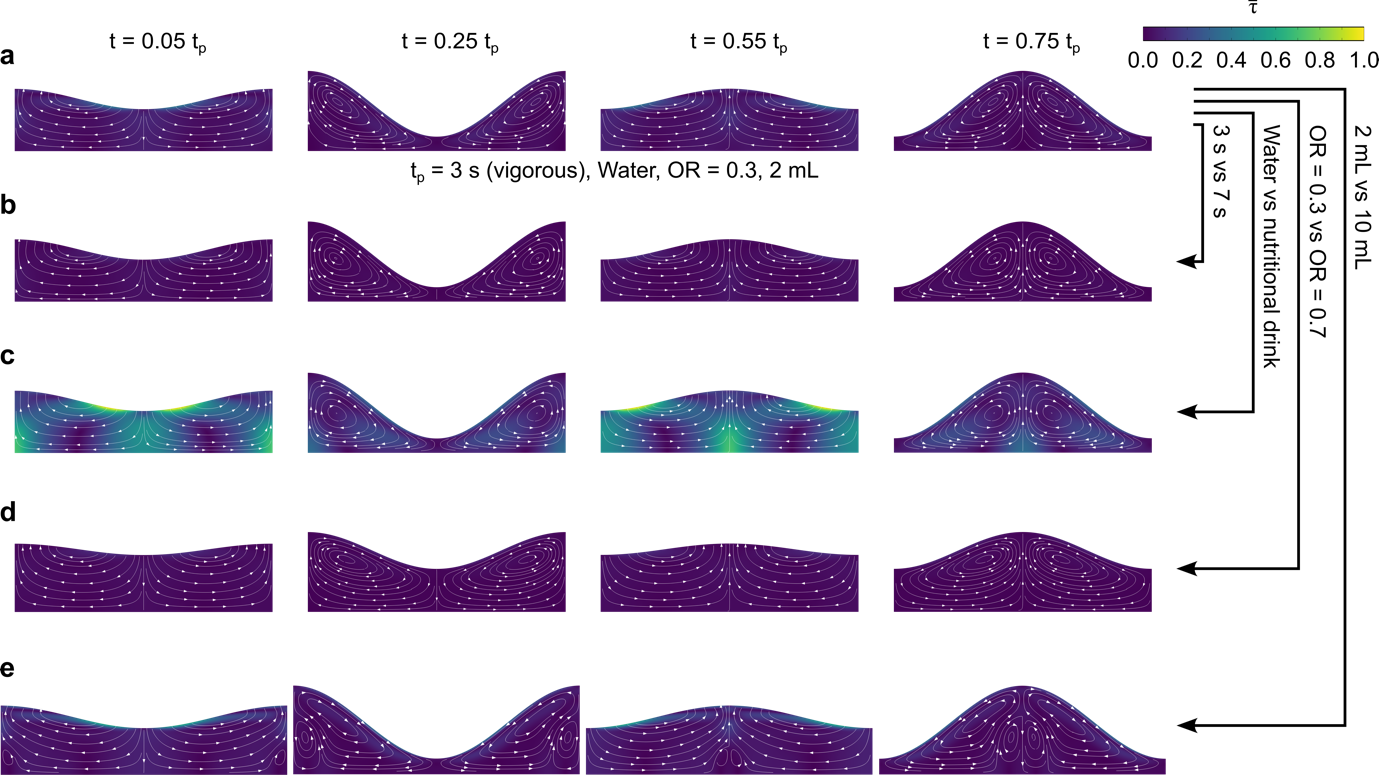


**Figure** **S1. Shear stress distributions for different variability factors in the segmentation at different time points. a** Baseline with vigorous intensity, water as the intestinal fluid, occlusion ratio (OR) of 0.3, and volume of 2 mL. **b** Light intensity. **c** Nutritional drink. **d** OR of 0.7. **e** Volume of 10 mL.


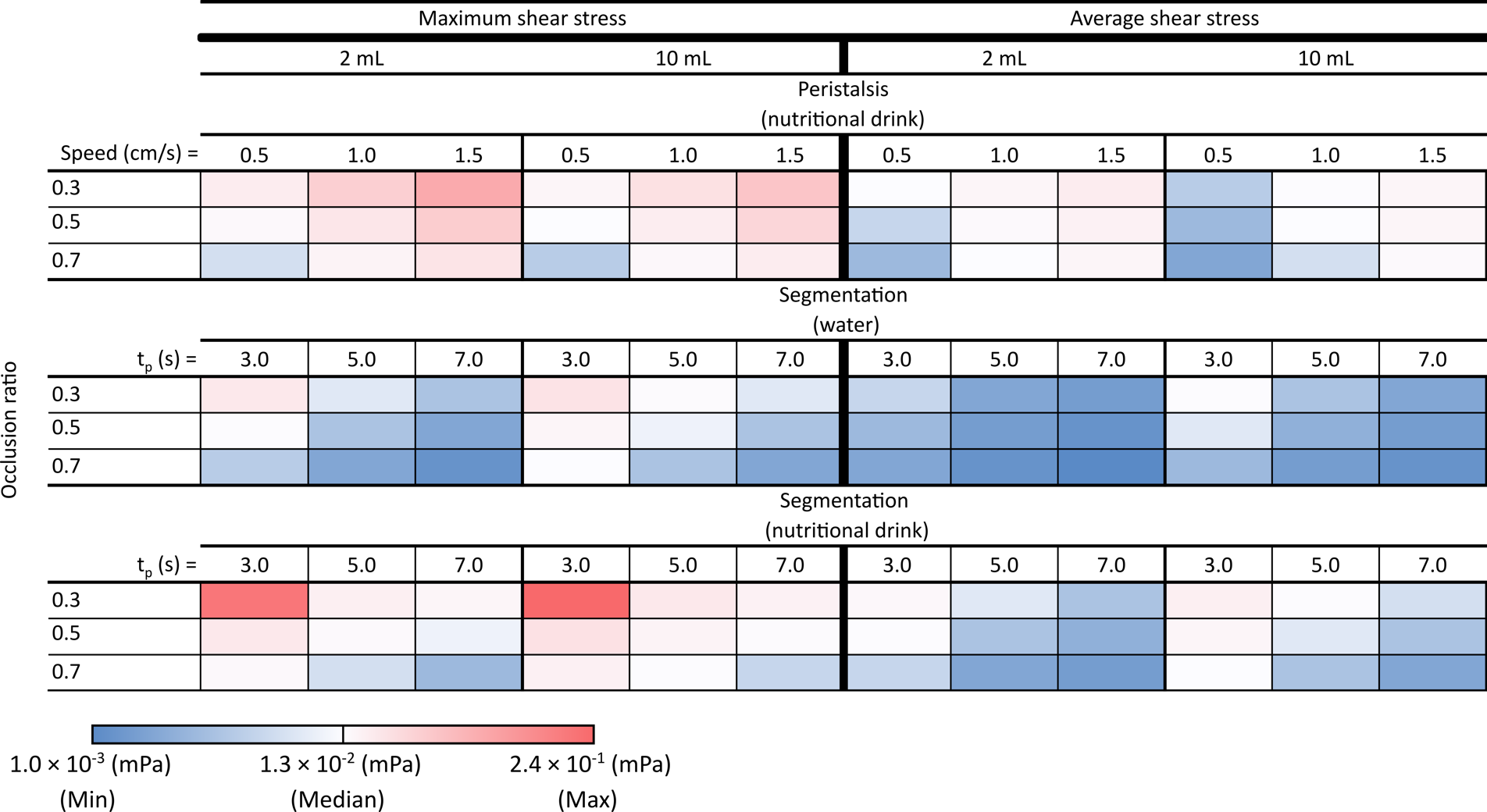


**Figure** **S2. Heat map of the maximum and average shear stresses at the epithelial surface for the cases in this study**

## Appendix C – Macromolecular concentration at the epithelium

Figure S3 shows the maximum normalised concentration values of insulin at the epithelial surface for all simulated cases. The data corroborated controlled release at the most occluding positions for cases with lower volume, light motility, and nutritional drink.


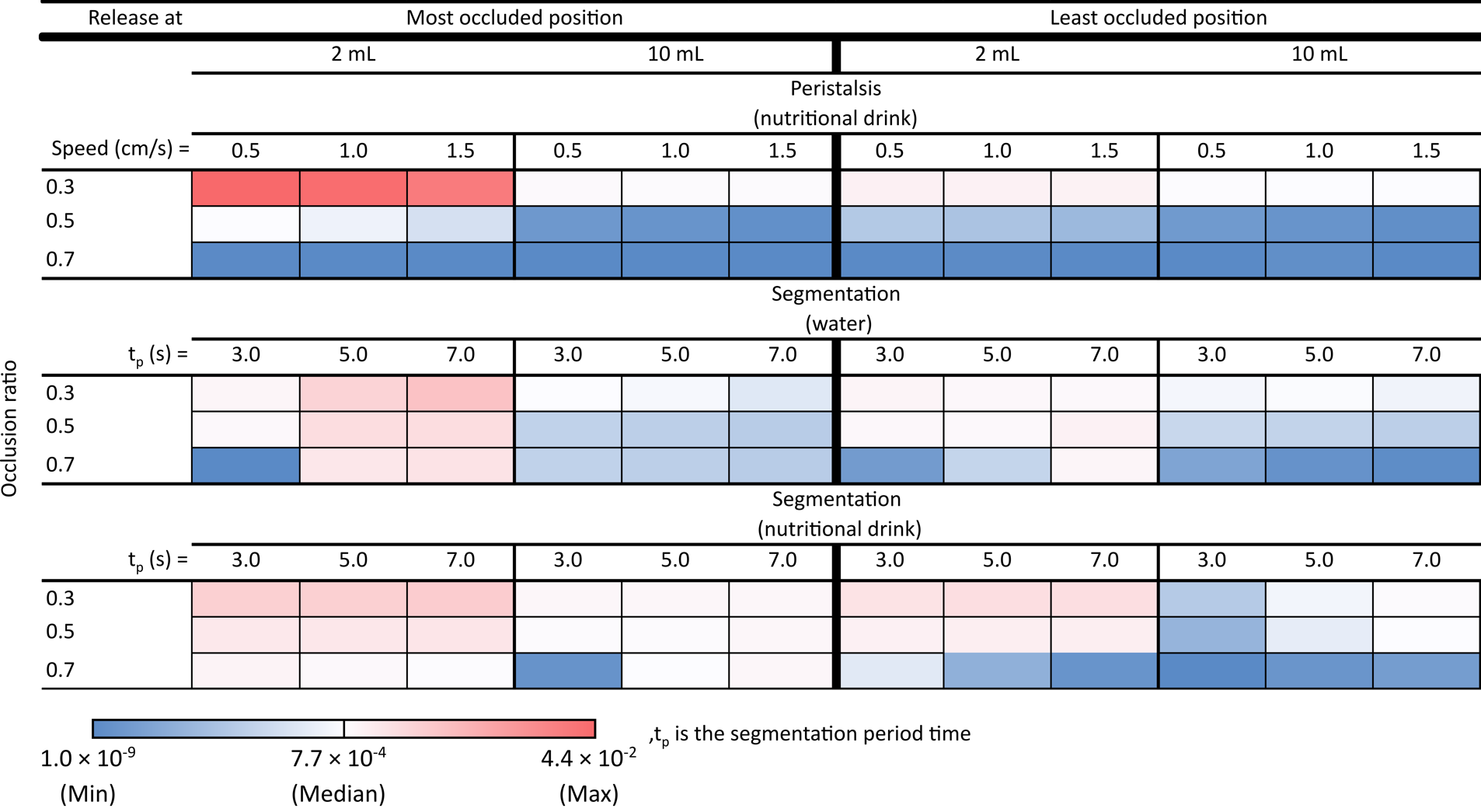


**Figure S3. Heat map of maximum normalised insulin concentration at the epithelial surface over the course of the simulation**

## Appendix D – Rheology measurements

Figures S4 and S5 present the experiments conducted to determine the rheological properties of the MiniMax nutritional drink. We fit a power-law model to capture the shear-thinning behaviour of the drink. Incorporating the model into the computations enabled us to input the specific dynamic viscosity at each element within the computational domain in accordance with the local shear rate value.


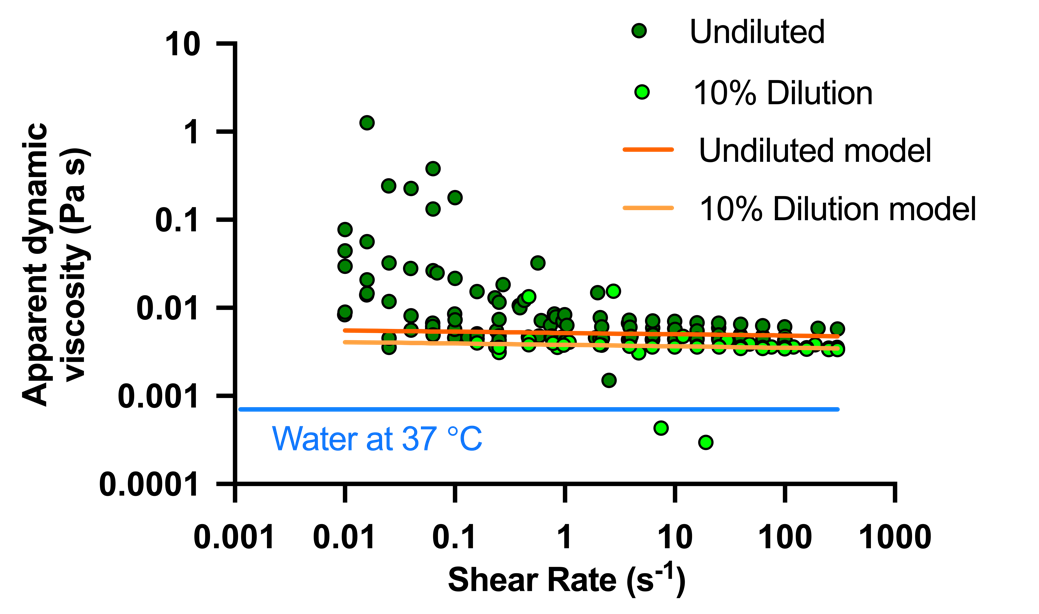


**Figure** **S4. Apparent dynamic viscosity (Pa s) of the nutritional drink MiniMax as a function of shear rate (0.01 s^-1^ to 300 s^-1^).** The viscosity of the nutritional drink was measured before and after dilution with 10% water. The green dots indicate the measured data points for both the undiluted and diluted samples. The orange lines represent the curve fits using a power-law formula. The blue line serves as a reference curve showing the baseline viscosity under standard conditions.


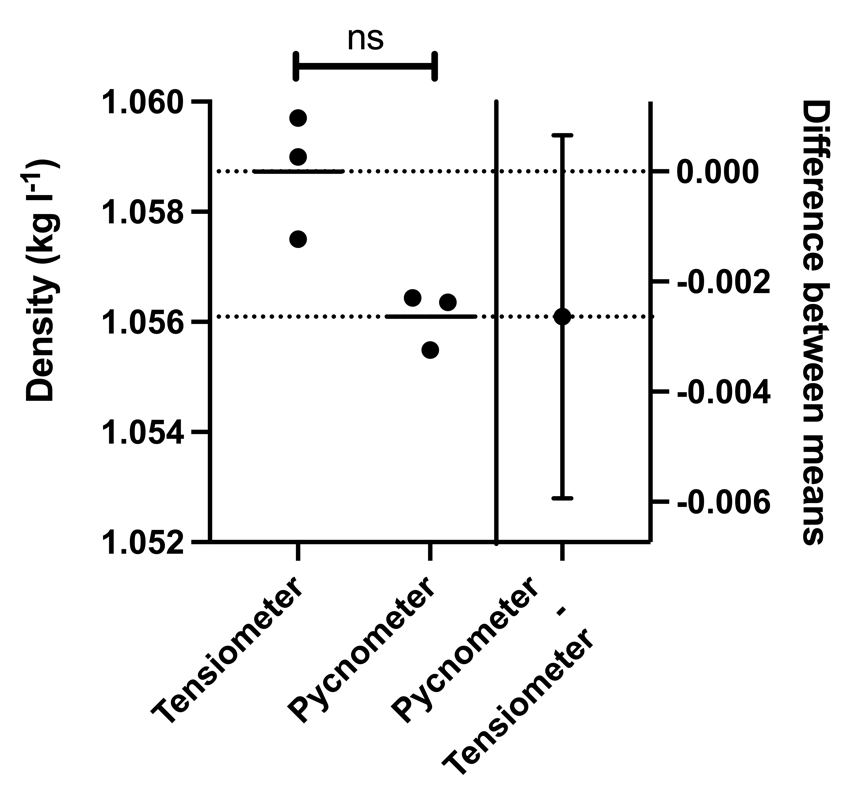


**Figure** **S5. Comparison of density measurements using a tensiometer and pycnometer for a solution of MiniMax diluted with 10% water.** The difference in mean density values between the two measurement methods was not statistically significant (unpaired t-test; P > 0.01).

## Appendix E – Grid sensitivity analysis for flow velocity components and macromolecule concentration

To ensure the accuracy of our CFD simulations, a grid sensitivity analysis was conducted that focused on the key elements of the momentum and mass transfer equations. Both the axial and radial velocity components were analysed to assess the impact of mesh refinement on the simulation results (Figure S6). The meshes used consisted of 2.73k, 5.32k, 10.31k, and 20.25k elements.

We compared the axial and radial components of the velocity for these four grids along the centreline and the radial line at the pocket centre (Figure S6a). This figure presents comparisons at various time points and highlights the consistency of the velocity profiles (Figure S6b,c). The consistency was particularly evident between the two finest meshes (10.31k and 20.25k elements), with curves in excellent agreement. This consistency indicates that both the axial and radial velocity components were effectively grid-independent at finer mesh resolutions.


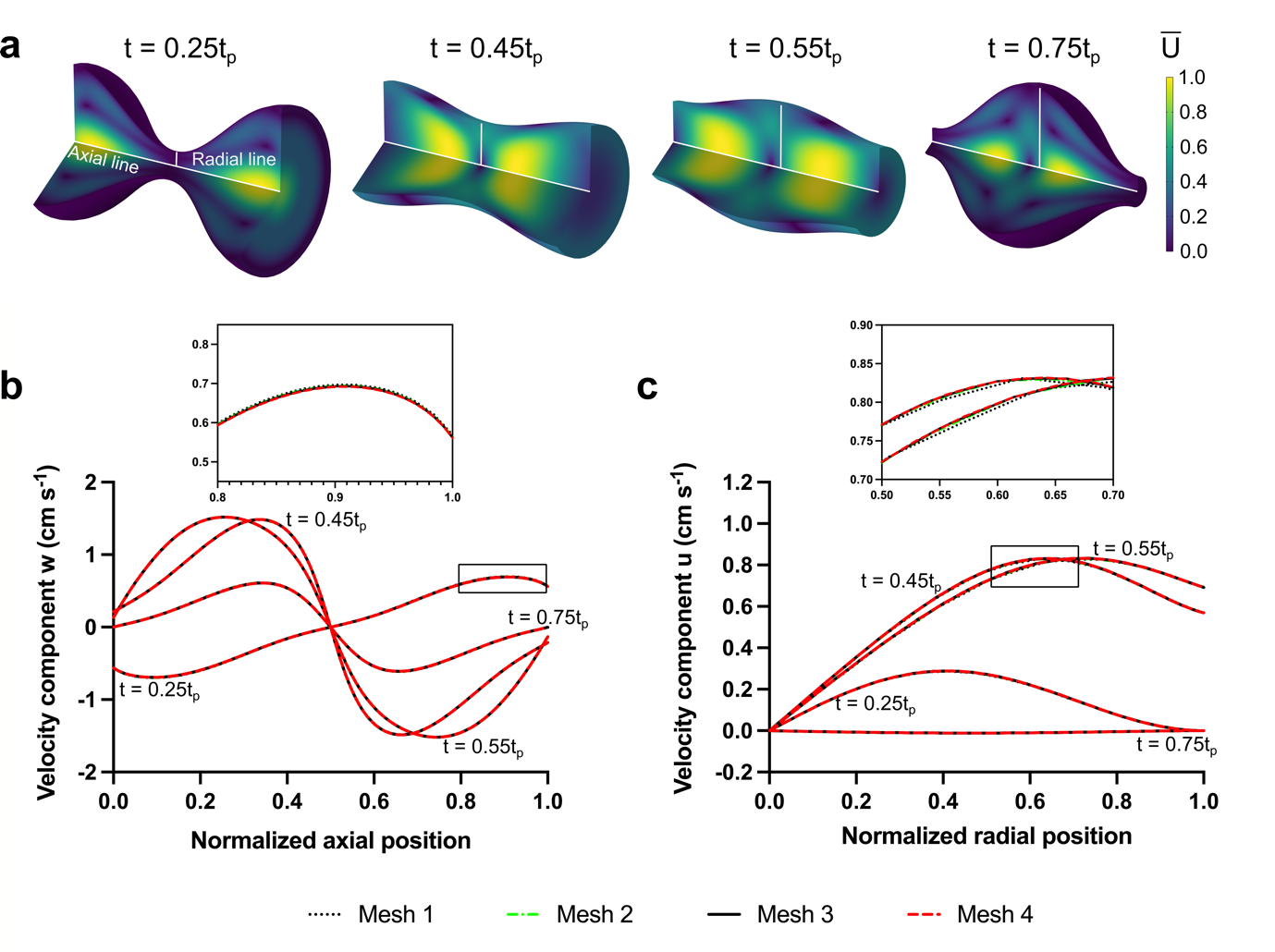


**Figure** **S6. Grid sensitivity analysis of the velocity components. a** Deformations of the pocket at selected time points coloured by the normalised velocity magnitude at vigorous intensity. **b** Axial component of velocity along the axial line for four different grids at different time points. **c** Radial component of the velocity along the radial line at the pocket centre.

We also extended the analysis to ensure that the mesh size did not affect the underlying mass transport. In particular, we monitored the average concentration of insulin at the epithelial surface over time for discrepancies that might have arisen from the different grid sizes. Supplementary Table 1 details the average differences in the concentrations between each two consecutive grids. A notable decrease (1.67%) in the average difference indicates reasonable convergence of the results.

**Supplementary Table 1. Average insulin concentration at the epithelial surface for generated meshes**

| Cases | Grid number | $\bar{c}$($t=10t_{p}$) $\times{10}^{-3}$ | $\bar{c}$($t=15t_{p}$) $\times{10}^{-3}$ | $\bar{c}$($t=20t_{p}$) $\times{10}^{-3}$ | $\bar{c}$ ($t=25t_{p}$) $\times{10}^{-3}$ | $\bar{c}$ ($t=30t_{p}$) $\times{10}^{-3}$ | Average difference (%) |
| --- | --- | --- | --- | --- | --- | --- | --- |
| Mesh 1 | 2.73k | 3.13 | 4.07 | 3.91 | 3.61 | 3.34 | - |
| Mesh 2 | 5.32k | 3.38 | 4.56 | 4.32 | 4.02 | 3.80 | 18.21 |
| Mesh 3 | 10.31k | 3.87 | 5.05 | 4.60 | 4.34 | 4.24 | 9.15 |
| Mesh 4 | 20.25k | 3.90 | 5.07 | 4.46 | 4.29 | 4.35 | 1.67 |

## Appendix F – Normality assessment of residuals for statistical analyses

To ensure the robustness of our statistical analyses, we conducted a normality evaluation for the residuals derived from each dataset. Normality was assessed to determine if parametric tests could be appropriately used or if non-parametric alternatives were required. The assessment involved both visual inspection and quantitative analysis. Figure S7 shows the Q-Q (Quantile-Quantile) plots of residuals from the statistical analyses of maximum concentration at the epithelial surface for both MM and PE. These Q-Q plots compare actual residuals (horizontal axis) with predicted residuals (vertical axis) for different conditions affecting intraluminal drug transport, including intensity of segmentation (light, moderate, and vigorous), fluid composition (water and nutritional drink), pocket volume (small and large), occlusion ratio (0.3, 0.5, and 0.7), release location (most occluded versus least occluded), and motility type (segmentation and peristalsis, Figure S7). The visual inspection of these plots shows significant deviations from the reference line which suggests the lack of normality across various conditions.

In addition to the visual inspection using Q-Q plots, Supplementary Table 2 provides the results of statistical normality tests for the residuals of each dataset for both MM and PE. Four normality tests were conducted including the D'Agostino-Pearson omnibus (K2), Anderson-Darling (A2*), Shapiro-Wilk (W), and Kolmogorov-Smirnov (distance) tests. Each test was applied to determine whether the data met the criteria for a normal distribution. The table includes detailed results for each variability factor (intensity, fluid composition, pocket volume, occlusion ratio, release location, and motility type). In all cases, the p-values were below the threshold (alpha = 0.05) which indicates a rejection of the null hypothesis of normality (Supplementary Table 2).

As indicated in the methods section, given the consistent failure of normality assessments, non-parametric statistical tests were employed throughout our analyses. The choice of non-parametric methods, such as Dunn's multiple comparisons test, the Mann-Whitney U test, and the Kruskal-Wallis test, was made to provide reliable conclusions that do not rely on the assumption of normality.


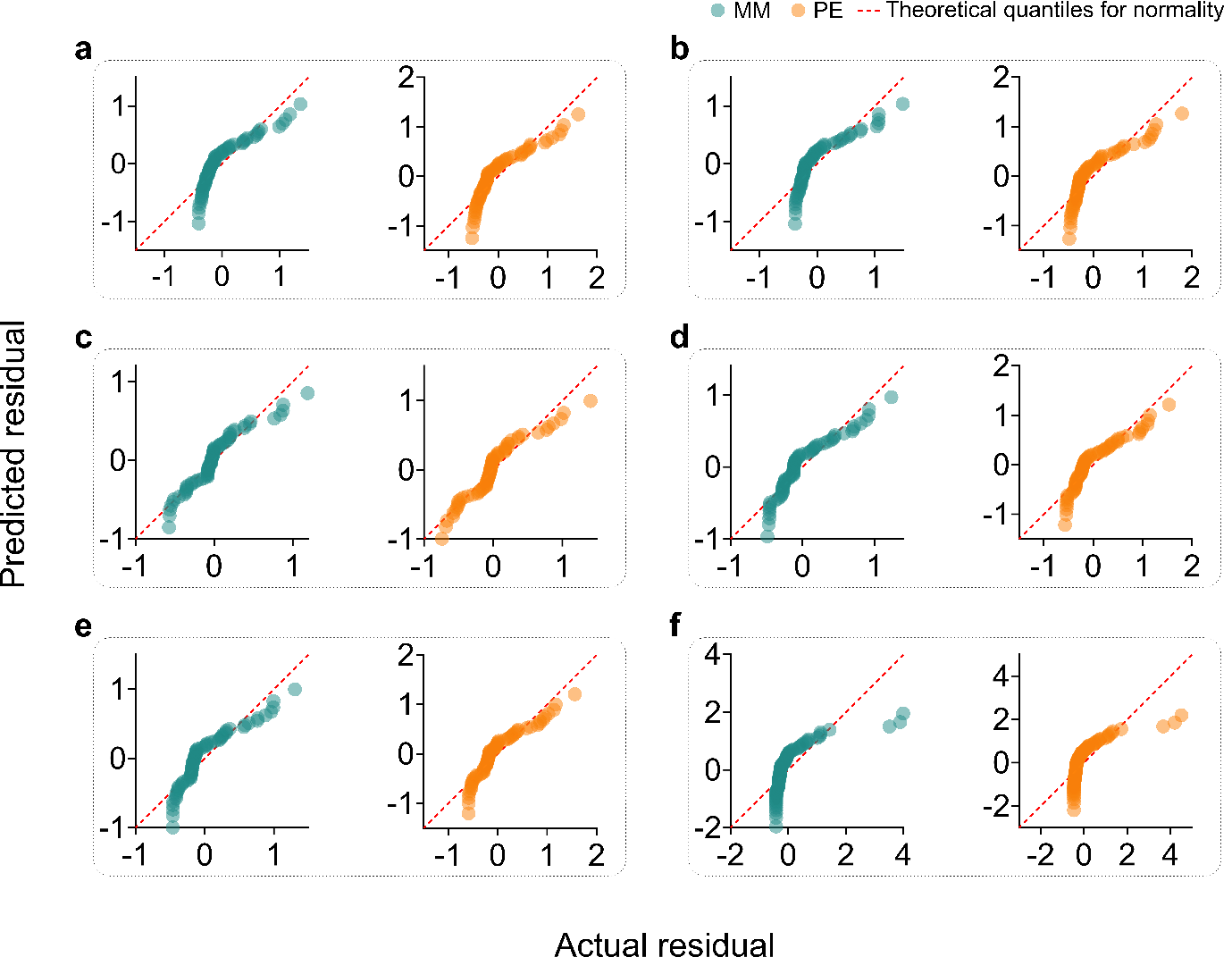


**Figure** **S7. Normality assessment of residuals from statistical analyses of maximum concentration at the epithelial surface for MM and PE.** Quantile–Quantile (QQ) plots compare observed residuals (horizontal axis) with predicted residuals (vertical axis) for the multifactor statistical analyses. **a** Light, moderate, and vigorous segmentation intensities. **b** Water and nutritional drink as luminal fluids. **c** Small (2 mL) and large (10 mL) pocket volumes. **d** OR values of 0.3, 0.5, and 0.7. **e** Release location at the most occluded and least occluded positions in the pocket. **f** Segmentation and peristalsis. An ordinary one-way ANOVA was used to derive QQ plots for cases involving three groups (e.g., intensity and occlusion ratio), whereas an unpaired t-test was used in cases with two groups (e.g., fluid type, motility type, pocket volume, and release location).

**Supplementary Table 2. Normality assessment of residuals for maximum concentration at the epithelial surface for MM and PE across various variability factors.**


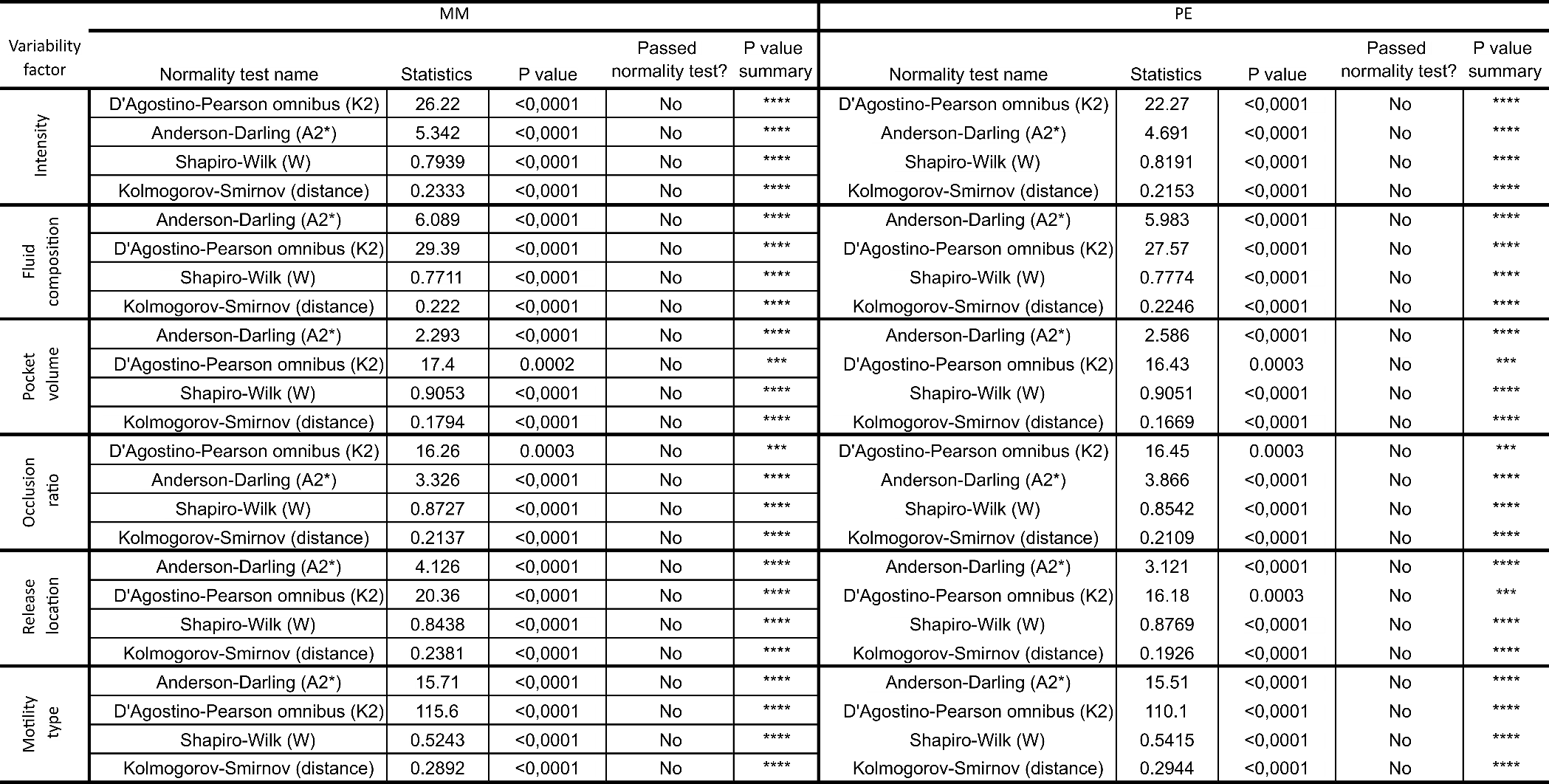

Supplement: Supplementary material — _Revised.docx [file IDRD_A_2607779_SM0405.docx]
